# First trimester pregnancy outcomes in a large IVF center from the Lombardy County (Italy) during the peak COVID-19 pandemic

**DOI:** 10.1038/s41598-021-96134-9

**Published:** 2021-08-16

**Authors:** P. E. Levi- Setti, F. Cirillo, V. Immediata, E. Morenghi, V. Canevisio, C. Ronchetti, A. Baggiani, E. Albani, P. Patrizio

**Affiliations:** 1 Department of Gynecology, Division of Gynecology and Reproductive Medicine, Fertility Center, Humanitas Research Hospital, IRCCS, Via Manzoni 56, 20089 Rozzano, Milan Italy; 2 Department of Obstetrics, Gynecology and Reproductive Sciences, School of Medicine, Yale University, New Haven, CT USA; 3 Biostatistics Unit, Humanitas Research Hospital, IRCCS, Rozzano, Milan Italy; 4 Department of Biomedical Sciences, Humanitas University, Via Rita Levi Montalcini 4, 20090 Pieve Emanuele, Milan Italy

**Keywords:** Diseases, Endocrinology, Health care

## Abstract

At the beginning of 2020, the Italian Lombardy region was hit by an “epidemic tsunami” which was, at that point in time, one of the worst pandemics ever. At that moment the effects of SARS-COV 2 were still unknown. To evaluate whether the pandemic has influenced ART (Assisted Reproduction Techniques) outcomes in an asymptomatic infertile population treated at one of the major COVID-19 epicentres during the weeks immediately preceding lockdown. All ART procedures performed during two time periods were compared: November 1st, 2018 to February 28th, 2019 (non-COVID-19 risk) and November 1st, 2019 to February 29th, 2020 (COVID-19 risk). In total 1749 fresh cycles (883 non-COVID-19 risk and 866 COVID-19 risk) and1166 embryos and 63 oocytes warming cycles (538 and 37 during non-COVID and 628 and 26 during COVID-19 risk, respectively) were analysed. Clinical pregnancies per cycle were not different: 370 (25.38%) in non-COVID versus 415 (27.30%) (*p* = 0.237) during COVID-19 risk. There were no differences in biochemical pregnancy rates 52 (3.57%) versus 38 (2.50%) (*p* = 0.089) nor in ectopic pregnancies 4 (1.08%) versus 3 (0.72%) (*p* = 0.594), spontaneous miscarriages 84 (22.70%) versus 103 (24.82%) *p* = 0.487, nor in intrauterine ongoing pregnancies 282 (76.22%) versus 309 (74.46%) *p* = 0.569. A multivariate analysis investigating differences in spontaneous miscarriage rate showed no differences between the two timeframes. Our results support no differences in asymptomatic infertile couples’ ART outcomes between the pre COVID and COVID-19 periods in one of the earliest and most severe pandemic areas.

## Introduction

At the beginning of 2020 the Italian Lombardy region was hit by an “epidemic tsunami”, one of the worst Severe Acute Respiratory Syndrome Coronavirus 2 (SARS-CoV-2) outbreaks to date^1^. Based on the first investigation on SARS-CoV-2 seroprevalence at the beginning of the outbreak in one of the two initial lockdown areas in Lombardy, i.e. Lodi, the virus exposure was detected in 28% of asymptomatic blood donors^2^. While the Lombardy region, which is home to 10 million inhabitants, accounted for 37% of cases and 53% of deaths in Italy as of 15th April 2020, the reported prevalence might have been underestimated by up to seven- ten times due to the non-availability at that time of nasopharyngeal swabs^2^. Although officially the first coronavirus disease (COVID-19) case was reported on February 21st at the Codogno (Lodi) Hospital located 57 km (about 35 miles) from our Fertility Center, a recent epidemiological analysis demonstrated that the SARS-CoV-2 was present in Northern Italy at least 3 months (end of December 2019) before the official recognition^3^. Therefore, it is highly plausible that during the months of January–February 2020, before the interruption of clinical services and the lockdown, several infertile patients who were asymptomatic and, thus, not known to be infected with SARS-CoV-2, were treated with ART (Assisted Reproduction Techniques) cycles and achieved pregnancies. This likely prolonged period of unmonitored community spread must be highlighted as key in the decision to undertake the analysis of ART outcomes in our region during this timeframe.

Available data on the impact of the SARS-CoV-2 on second and third trimester pregnancies such as preterm birth incidence^4^are still limited and contentious thus representing fertile soil for future research. Some authors suggested an increased risk for preterm deliveries^5^ and caesarean sections^6^ during the pandemic, also because of decreased patient-provider interactions. Others instead showed a decrease in premature births^7^ and suggested that this might be related to lifestyle modifications due to the lockdown, i.e. lower infection load and decreased physical activity, that are possibly beneficial for reducing extreme prematurity and infant mortality. There are no controversial data, on the other hand, that the risk for vertical transmission is minimal^8^, although some have reported a significantly higher stillbirth rate^9^ either as a possible indirect effect of the reluctance to go to the hospital even when necessary, or secondary to staff shortages or to reduced antenatal visits.

Case series have reported the detection of SARS-CoV-2 in various biological material, from semen to human breastmilk^10–12^, and the possible vertical transmission of the virus from an infected mother to her newborn^13^ raising serious concerns in the embryology community worldwide^14,15^. The mother-to-child transmission of SARS-CoV-2 remains a matter of open debate and, as Mahyuddin et al. concluded in their review^16^, “further spatial‐temporal studies are needed to determine the true potential for transplacental migration”.

Moreover, there is very limited information on the possible consequences of SARS-CoV-2 infection on ART performance and early pregnancy outcome.

Several cross-sectional studies have shown a high rate of asymptomatic carriages amongst pregnant women. The universal use of SARS-CoV-2 testing in all pregnant patients presenting for delivery revealed that during the pandemic in New York City^17^, most of the patients positive for SARS-CoV-2 at delivery were asymptomatic.

Thus, there is a high plausibility that women even during the early stages of their pregnancy may have had asymptomatic infections^18^. The aim of this work is to evaluate early pregnancy outcomes after assisted reproduction during the early peak of COVID-19 in Lombardy. Despite the difficulty in determining the extent of asymptomatic SARS-CoV-2 infection due to lack of testing, we compared a cohort of infertile patients undergoing ART during high COVID-19 risk (November 2019 to February 29, 2020) with a cohort treated during no COVID-19 risk (November 2018 to February 28, 2019) and evaluated for differences in ART and early pregnancy outcomes.

## Material and methods

This is a retrospective observational analysis from a single, tertiary care, university-affiliated fertility centre, located in Lombardy, Rozzano, Milan, Italy, including all couples who underwent Assisted Reproduction (ART) procedures (fresh and frozen transfer cycles) during two time periods used for comparison: November 1st, 2018 to February 28th, 2019 (non-COVID-19 risk, timeframe A) and November 1st, 2019 to February 29th, 2020 (COVID-19 risk, timeframe B).

No exclusion criteria were considered. The entirety of patients was from all over Italy, with 70% from the Lombardy region. The great majority of patients spent the entire period of the ovarian stimulation/monitoring within-2 h car distance from the hospital.

Information collected included: female age, BMI and smoking habits, duration of infertility, basal Follicle Stimulating Hormone (FSH), Anti Mullerian Hormone (AMH), Antral Follicular Count (AFC), indication for ART treatment, primary or secondary infertility and previous miscarriages. In addition, follow up data on pregnancy, first trimester spontaneous miscarriages, and ectopic pregnancy outcome were collected until June 15, 2020 (representing the end of the 1st trimester of the last included patient).

Testing data for SARS-CoV-2 was not always available and therefore was not reviewed. Of note, one embryologist with very mild symptoms had a confirmed positive oropharyngeal swab for SARS-CoV-2 during the study period, but no one else among medical, embryological, nurse and staff (about 60 people) had symptoms relatable to the viral infection.

Patient follow-up included beta-hCG level 12 days after blastocyst transfer or 14 days after cleavage stage transfer, repeated every 48 h for pregnant patients , until reaching at least 1500 UI/ml. Transvaginal ultrasound was scheduled 4 weeks after transfer or earlier in case of abdominal pain and vaginal bleeding or abnormal rising beta hCG levels. All non-essential ART activities were interrupted on April 16, 2020. Only emergency services, including fertility preservation for oncological patients and pregnancy follow-ups were allowed during the lockdown period.

Patients were required to email the results of ultrasound if performed in other facilities. Every day, medical staff and assistants on duty called patients and updated the pregnancy outcome in their medical charts. Less than 5% of patients were lost to follow-up, which was concluded upon completion of 12-weeks-gestation, i.e. the end of 1st trimester (as of June 15, 2020).

Clinical pregnancy was defined as a pregnancy diagnosed by ultrasonographic visualization of one or more gestational sacs or definitive clinical signs of pregnancy^19^. Pregnancy rate is the number of clinical pregnancies on the number of procedures performed. It therefore includes ectopic pregnancies. The implantation rate is the number of intrauterine gestational sacs on the number of embryos transferred^19^.

Pregnancies with a Beta-hCG level peaking under 1000 UI/ml and with exclusion of extrauterine pregnancies were considered "biochemical".

### Statistical analysis and variable description

Data were described as number and percentage, or mean and standard deviation, as appropriate. Associations with period (pre and during COVID) were explored with χ2 test for categorical variables, or t student test for Gaussian continuous variables, or Mann Whitney for asymmetrical continuous variables. Association with spontaneous miscarriage rate was explored using logistic regression analysis. Independent variable with a p value under 0.2 were then submitted to a multivariable logistic regression analysis. Pre and during COVID risk, variables were included in the multivariable analysis being the primary endpoint of the research. A p value under 0.05 was considered as significant. All analyses were conducted using Stata Statistical Software: Release15 (College Station, TX: StataCorp LLC).

### Ethical approval

Generally, all patients undergoing ART procedures consent in writing that their medical records can be used for research purposes if anonymity and confidentiality is protected. Since both conditions were met, this study had expedited review and approval by the center’s Institutional Review Board (IRB), Humanitas Clinical Institute Ethic Committee.

All methods were performed in accordance with the relevant guidelines and regulations and the present research was performed in accordance with the Declaration of Helsinki.

An informed specific consent was obtained from all participants and/or their legal guardians.

The study was also approved by our Independent Ethical Committee on May 14th, 2020 (protocol n. 37/20).

## Results

A total of 2978 cycles were analyzed, 1458 during timeframe A (controls) and 1520 during timeframe B (exposure). Of these, 1749 (58.70%) were fresh cycles, 883 in the period from November 1st, 2018 to February 28th, 2019 (timeframe A, controls) and 866 in the period November 1st, 2019 to February 29th, 2020 (timeframe B, risk exposure). A total of 1166 (39.10%) embryo warming cycles, 538 during non-COVID-19 and 628 during COVID-19 risk and, 63 (2.20%) oocytes warming cycles, 37 in the non-COVID period and 26 during the COVID-19 risk were also assessed (see Table 1).

Female age was 37.11 ± 4.17 years in timeframe A and 36.56 ± 4.19 in timeframe B (*p* = 0.026). No differences were found in other patient’s demographic, ovarian response, and laboratory parameters (see Table 1). The number of transfers per started cycle, number of embryos transferred and the freeze-all cycles were the only variables significantly different between the two periods (as shown in Table 1).

**Table 1:** Patients, demographics, indications for ART and cycle characteristics for the total fresh cycles analyzed in the non-COVID (November 1st, 2018 to February 28th, 2019) and COVID-19 (November 1st, 2019 to February 29th, 2020) timeframes.

Fresh cycles	non-COVID	COVID-19	*p*
N	883	866	
Female age	37.11 ± 4.17	36.56 ± 4.19	0.026
Years of infertility	3.8 ± 2.6	3.72 ± 2.45	0.734
BMI	22.26 ± 3.34	22.41 ± 3.37	0.299
Smoking	146 (16.53%)	177 (20.44%)	0.035
Secondary infertility	312 (35.33%)	272 (31.41%)	0.082
Previous deliveries	41 (4.64%)	28 (3.23%)	0.130
Previous miscarriages	275 (31.14%)	234 (27.02%)	0.058
Basal FSH	8.55 ± 3.61	8.67 ± 3.59	0.313
AMH	2.46 ± 2.49	2.5 ± 2.65	0.702
Antral follicular count	11.24 ± 8.11	11.72 ± 8.51	0.396
**Indication to treatment**			
Male	227 (25.71%)	239 (27.60%)	0.371
Tubal	55 (6.23%)	36 (4.16%)	0.051
Unexplained	103 (11.66%)	86 (9.93%)	0.243
Male and female factor	214 (24.24%)	207 (23.90%)	0.871
Ovulatory	11 (1.25%)	11 (1.27%)	0.963
Reduced ovarian reserve	183 (20.72%)	138 (15.94%)	0.010
Multiple female factors	49 (5.55%)	35 (4.04%)	0.140
Other	14 (1.59%)	8 (0.92%)	0.214
**Stimulation protocol**			0.320
Antagonist cycle	692 (78.37%)	665 (76.79%)	
Agonist long short	46 (5.21%)	60 (6.93%)	
Agonist flare	145 (16.42%)	141 (16.28%)	
Total sperm count (× 10^6^)	74.07 ± 90.82	71.85 ± 76.31	0.393
Progressive motility %	23.59 ± 9.87	24.6 ± 11.56	0.221
Oocyte retrievals	820	783	0.064
Cycles Cancelled	63 (7.13%)	83 (9.58%)	0.064
Mean Oocytes retrieved	9.32 ± 6.06	10.05 ± 6.64	0.070
No oocytes retrieved or not usable	18 (2.04%)	14 (1.62%)	0.510
Mature Oocytes	6.98 ± 4.93	7.35 ± 5.29	0.376
Fertilization rate %	76.66 ± 23.56	74.52 ± 24.75	0.113
Cleavage rate %	98.63 ± 7.64	98.86 ± 5.22	0.879
Number of transfers per started cycles	567/883 (64.21%)	496/866 (57.27%)	0.003
Mean Embryos transferred	1.72 ± 0.51	1.64 ± 0.5	0.010
Cleavage stage	473 (83.42%)	404 (81.45%)	
Blastocyst stage	94 (16.58%)	92 (18.55%)	
Frozen embryos	2.39 ± 1.46	2.59 ± 1.62	0.097
Frozen Oocytes	8.19 ± 4.08	8.3 ± 4.01	0.887
Freeze All Cycles	224 (27.32%)	257 (32.82%)	0.016

Cycle outcomes are described in Table 2. In the fresh cycles, implantation rates, biochemical pregnancies, clinical pregnancies per cycle and per transfer, ectopic pregnancies, spontaneous miscarriages and intrauterine ongoing pregnancies were not significantly different between the two periods analyzed (Table 2).

**Table 2:** ART cycles outcomes in fresh, warmed embryos and warmed oocytes in the non-COVID and COVID-19 timeframes.

	non- COVID	COVID	*p*
**Fresh embryo transfers**			
N	883	866	
Female age	37.11 ± 4.17	36.56 ± 4.19	0.026
Embryo transferred	1.72 ± 0.51	1.64 ± 0.5	0.010
Cleavage stage	473 (83.42%)	404 (81.45%)	0
Blastocyst stage	94 (16,58%)	92 (18,55%)	0.399
Implantation rate %	24.02 ± 38.95	28.39 ± 39.54	0.079
Biochemical Pregnancies	26 (2.94%)	13 (1.50%)	0.041
Clinical Pregnancies per cycle	189 (21.40%)	188 (21.71%)	0.877
Clinical Pregnancies per transfer	189 (33.33%)	188 (37.90%)	0.120
Ectopic Pregnancies	3 (1.59%)	3 (1.60%)	1.000
Miscarriages	37 (19.58%)	50 (26.60%)	0.106
Intrauterine ongoing pregnancies	149 (78.84%)	135 (71.81%)	0.114
Single pregnancies	127 (85.23%)	112 (82.96%)	0.601
Twin pregnancies	22 (14.77%)	23 (17.04%)	
**Embryo warmings**			
N	538	628	
Female age at freezing	29.6 ± 13.8	28.9 ± 14.7	0.782
Warmed embryos	1.058 ± 0.23	1.053 ± 0.24	0.565
Survived embryos	1.051 ± 0.22	1.043 ± 0.25	0.430
Embryo transferred	1.049 ± 0.22	1.036 ± 0.23	0.285
Cleavage stage	32 (7.14%)	23 (4,58%)	0.091
Blastocyst stage	409 (91.29%)	464 (92,43%)	
Embryos from other institutions	7 (1.56%)	15 (2,99%)	0.195
Implantation rate %	38.95 ± 49.89	44.22 ± 50.71	0.100
Biochemical Pregnancies	25 (4.65%)	23 (3,66%)	0.399
Clinical Pregnancies per cycle	174 (32.34%)	222 (35.35%)	0.280
Clinical Pregnancies per transfer	174 (38.84%)	222 (44.22%)	0.093
Ectopic Pregnancies	1 (0.57%)	0 (0.00%)	0.439
Miscarriages	45 (25.86%)	53 (23.87%)	0.649
Intrauterine ongoing pregnancies	128 (73.56%)	169 (76.13%)	0.559
Single pregnancies	127 (99.22)	164 (97.04%)	0.187
Twin pregnancies	1 (0.78%)	5 (2.96%)	
**Oocyte warmings**			
N	37	26	
Female age at freezing	30.2 ± 12.4	25.9 ± 15.0	0.159
Warmed oocytes	6.66 ± 2.62	6.9 ± 2.34	0.629
Survived oocytes	4.94 ± 2.54	5.85 ± 1.84	0.156
Fertilized oocytes	4 ± 2.48	4.5 ± 1.76	0.482
Transferred embryos	1.68 ± 0.6	1.75 ± 0.55	0.639
Cleavage stage	29 (100.00%)	18 (94.74%)	0.396
Blastocyst stage	0 (0.00%)	1 (5.26%)	
Pregnancies	7	5	
Implantation rate %	18.97 ± 36.39	13.16 ± 22.62	0.911
Biochemical Pregnancies	1 (2.70%)	2 (7.69%)	0.564
Clinical Pregnancies per cycle	7 (18.92%)	5 (19.23%)	0.975
Clinical Pregnancies per transfer	7 (24.14%)	5 (26.32%)	0.865
Ectopic Pregnancies	0	0	
Miscarriages	2 (28.57%)	0 (0.00%)	0.470
Intrauterine ongoing pregnancies	5 (71.43%)	5 (100%)	0.470
Single pregnancies	3 (60.00%)	5 (100%)	0.444
Twin pregnancies	2 (40.00%)	0	

A total of 2061 (69.21%) IVF cycles involved embryo transfer during the respective timeframes, 1044 in the non-COVID (71.60%) and 1017 (66.10%) during the COVID-19 risk period (*p* = 0.005). Pregnancies per cycle started were 370 (25.38%) versus 415 (27.30%) (*p* = 0.237); pregnancies in fresh cycles were 189 (21.40%) versus 188 (21.68%) (*p* = 0.887); pregnancies in frozen embryos were 174 (32.34%) versus 222 (35.35%) (*p* = 0.280). Pregnancies in frozen oocytes cycles were 7 (18.92%) versus 5 (19.23%) (*p* = 0.975) in the two timeframes, respectively. The overall implantation rate in percentage was 30.28 ± 43.59 versus 35.92 ± 45.93 (*p* = 0.006), but it was not significantly different between fresh and frozen cycles. In fresh cycles it was 24.02 ± 38.95 versus 28.39 ± 39.54 *p* = 0.079 while in frozen embryo transfer cycles it was 38.95 ± 49.89 versus 44.22 ± 50.71 (*p* = 0.100) for the non-COVID and the COVID-19 risk, respectively. The use of frozen oocytes cycles had an implantation rate of 18.97 ± 36.39 versus 13.16 ± 22.62, *p* = 0.911 for the controls and risk exposure, respectively. Biochemical pregnancies were 52 (3.57%) versus 38 (2.50%) (*p* = 0.089), clinical pregnancies per cycle 370 (25.38%) versus 415 (27%) (*p* = 0.237), ectopic pregnancies 4 (1.08%) versus 3 (0.72%) (*p* = 0.594), spontaneous miscarriages 84 (22.70%) versus 103 (24.82%) (*p* = 0.487), intrauterine ongoing pregnancies 282 (76.22%) versus 309 (74.46%) (*p* = 0.569) (Table 3).

**Table 3:** Summary ART cycles outcome in the total population during pre COVID and COVID-19 timeframes.

Variable	non-COVID	COVID-19	p
Total cycles (n)	1458	1520	
Fresh Cycles	883 (57.13%)	866 (56.94%)	
Frozen Embryos	538 (26.90%)	628 (41.29%)	
Frozen Oocytes	37 (2.54%)	26 (1.71%)	
Total transfers (n)	1044 (71.60%)	1017 (66.91%)	0.005
Pregnancies (% per cycle)	370 (25.38%)	415 (27.30%)	0.237
Pregnancies fresh cycles (% per cycle)	189 (21.40%)	188 (21.68%)	0.887
Pregnancies frozen embryos (% per cycle)	174 (32.34%)	222 (35.35%)	0.280
Pregnancies frozen oocytes (% per cycle)	7 (18.92%)	5 (19.23%)	0.975
Implantation rate	30.28 ± 43.59	35.92 ± 45.93	0.006
Implantation rate fresh cycles	24.02 ± 38.95	28.39 ± 39.54	0.079
Implantation rate frozen embryos	38.95 ± 49.89	44.22 ± 50.71	0.100
Implantation rate frozen oocytes	18.97 ± 36.39	13.16 ± 22.62	0.911
Biochemical Pregnancies	52 (3.57%)	38 (2.50%)	0.089
Clinical Pregnancies (% per cycle)	370 (25.38%)	415 (27,30%)	0.237
Ectopic Pregnancies	4 (1.08%)	3 (0.72%)	0.594
Miscarriages	84 (22.70%)	103 (24.82%)	0.487
Intrauterine ongoing pregnancies	282 (76.22%)	309 (74.46%)	0.569
Single pregnancies	257 (91.13%)	281 (90.94%)	0.934
Twin pregnancies	25 (8.87%)	28 (9.06%)	

A multivariate analysis investigating differences in the spontaneous miscarriage rate (Table 4) between the COVID and non-COVID-19 period showed no differences. There were 103 versus 312 spontaneous miscarriages, with no difference between the two timeframes analyzed at the univariate analysis (*p* = 0.487, OR 1.12 (0.81–1.56) and at the final multivariate model (*p* = 0.953 OR 0.99 (0.68–1.44). The only variables significantly related to a higher miscarriage rate were female age (1.11 (1.06–1.17) *p* =  < 0.001), secondary infertility (0.57 (0.37–0.89), *p* = 0.014), previous miscarriages (9.50 (6.09–14.81) *p* =  < 0.001) and unexplained infertility (0.42 (0.23–0.76), *p* = 0.004).

**Table 4:** Univariate and multivariate analysis for variables potentially related to miscarriage rate in the analyzed population.

	Miscarriage	Not Miscarriage	*p*	Univariate		Multivariate final model	
				OR	*p*	OR	*p*
N	187	598					
COVID- non-COVID-19 risk	103 (55.08%)	312 (52.17%)	0.487	1.12 (0.81–1.56)	0.487	0.99 (0.68–1.44)	0.953
Female age	37.30 ± 4.18	35.53 ± 3.86	< 0.001	1.12 (1.08–1.19)	< 0.001	1.11 (1.06–1.17)	< 0.001
Years of infertility	4.28 ± 2.74	3.97 ± 2.53	0.3602	1.04 (0.98–1.11)	0.164		
BMI	21.85 ± 3.00	21.93 ± 3.07	0.9191	0.99 (0.94–1.04)	0.748		
Smoking	43 (22.99%)	122 (20.40%)	0.447	1.17 (0.79–1.73)	0.448		
Secondary infertility	91 (48.66%)	200 (33.44%)	< 0.001	1.89 (1.35–2.63)	< 0.001	0.57 (0.37–0.89)	0.014
Previous deliveries	13 (6.95%)	33 (5.52%)	0.466	1.28 (0.66–2.48)	0.467		
Previous miscarriages	131 (70.05%)	144 (24.08%)	< 0.001	7.38 (5.12–10.62)	< 0.001	9.50 (6.09–14.81)	< 0.001
Basal FSH	7.93 ± 2.72	8.06 ± 3.94	0.7155	0.99 (0.94–1.04)	0.679		
AMH	2.95 ± 2.56	3.16 ± 2.94	0.2795	0.97 (0.91–1.03)	0.377		
Antral follicular count	13.13 ± 10.42	14.12 ± 9.32	0.0439	0.99 (0.97–1.01)	0.325		
**Indication to treatment**							
Male	65 (34.76%)	233 (38.96%)	0.301	0.83 (0.59–1.18)	0.302		
Tubal	9 (4.81%)	36 (6.02%)	0.535	0.79 (0.37–1.67)	0.536		
Unexplained	18 (9.63%)	81 (13.55%)	0.159	0.68 (0.40–1.17)	0.161	0.42 (0.23–0.76)	0.004
Male and female factor	38 (20.32%)	118 (19.73%)	0.860	1.04 (0.69–1.56)	0.860		
Ovulatory	4 (2.14%)	6 (1.00%)	0.227	2.16 (0.60–7.73)	0.238		
Reduced ovarian reserve	15 (8.02%)	54 (9.03%)	0.671	0.88 (0.48–1.60)	0.671		
Multiple female factors	11 (5.88%)	19 (3.18%)	0.092	1.90 (0.89–4.08)	0.097		
Other	0	4 (0.67%)	0.262	NC			
Embryos transferred	1.37 ± 0.52	1.36 ± 0.48	0.9105	1.06 (0.76–1.49)	0.718		
Procedure (fresh)	87 (46.52%)	290 (48.49%)	0.638	0.92 (0.66–1.28)	0.638		

## Discussion

The results of our analysis showed no differences in early pregnancy outcomes between the controls (patients treated during non-COVID 19 risk) and patients treated during high COVID-19 risk exposure in terms of implantation, pregnancy, biochemical and spontaneous miscarriage rate both in fresh and frozen ART cycles. The lack of an increased risk for miscarriage or other adverse outcome in early pregnancies obtained during high risk COVID-19 exposure is quite reassuring. Plausible explanation for this is that probably asymptomatic exposure to the virus had no systemic effects. In addition, female age was one year younger for patients treated during the timeframe B (*p* = 0.026) and the miscarriage rate is known to be lower among young women. It is also possible that the younger population was less affected by the first wave of the SARS-CoV-2 pandemic.

Preliminary studies on pregnant women infected by SARS-CoV-2 gave an optimistic outlook regarding the clinical course of women who underwent ART procedures^20^ though recent studies suggest that pregnant women might be at increased risk for severe illness associated with COVID-19^21,22^. Zambrano and colleagues, members of the Center of Disease Control and Prevention (CDC) COVID-19 Response Pregnancy and Infant Linked Outcomes Team, showed how pregnant women were significantly more likely than non-pregnant women to be admitted to an intensive care unit (ICU) (10.5 vs. 3.9 per 1000 cases; adjusted risk ratio [aRR] = 3.0; 95% confidence interval [CI] = 2.6–3.4), to receive invasive ventilation (2.9 vs. 1.1 per 1000 cases; aRR = 2.9; 95% CI = 2.2–3.8), extracorporeal membrane oxygenation (ECMO) (0.7 vs. 0.3 per 1000 cases; aRR = 2.4; 95% CI = 1.5–4.0), and die (1.5 vs. 1.2 per 1000 cases; aRR = 1.7; 95% CI = 1.2–2.4)^23^. Due to this evidence and preliminary findings of mRNA COVID-19 vaccine safety in pregnant women^24^, several women's health scientific societies have endorsed vaccination in pregnant populations as they are considered at risk of severe COVID-19^25^. In fact, the frequency of adverse pregnancy and perinatal outcomes amongst vaccinated pregnant women is similar to pre-COVID baseline rates.

The World Health Organization on 11^th^ March 2020 declared the pandemic status for COVID-19, but Italy, mostly in the northern region of Lombardy, had already begun experiencing the severity of COVID-19 since February 21, 2020, or even before. To prevent the diffusion of contagion and avoid overwhelming the healthcare system, as well as to reduce the anxiety of establishing a pregnancy during uncertain times, on March 17, 2020 the Italian National Institute of Health (ISS) and the National Center of Transplants (CNT) issued preventive measures against the transmission of the new Coronavirus infection (SARS-CoV-2) by mandating interruption of all non-essential medical services, including ART-related procedures^42^. In view of the unknown effects of SARS-CoV-2 on mothers and fetuses, other international human reproduction societies published guidelines for managing patients who were already in an ART cycle or planning to start treatment. On March 19, 2020, the European Society for Human Reproduction and Embryology (ESHRE) established to defer new pregnancies from embryo transfers and to reduce the burden on health care workload by avoiding non-emergent hospital admissions. After six days a ‘COVID-19 working group’ met in order to publish new ART guidelines after reviewing the latest scientific reports^15^. On March 17, 2020, the document named “Patient Management and Clinical Recommendations During the Coronavirus (COVID-19) Pandemic” published by ASRM recommended to suspend all new ART cycles and all embryo transfers, providing help and care only for patients currently “in-cycle” or who needed urgent stimulation and cryopreservation. The intent was to suspend all non-urgent surgery and diagnostic procedures in order to minimize in-person interactions. Other updates followed over the weeks^25^.

These national and international guidelines were issued to safeguard the health of ART operators as well as of couples undergoing ART and newborns, during the COVID-19 pandemic^32^. From March 2020 to date, more than a year passed and many changes occurred. The abovementioned guidelines were formulated when there was still little understanding, great fear for a few cases of very sick pregnant women and a policy to redirect resources seen as non-necessary and with lower priority, such those for fertility treatment. Nowadays knowledge has increased, and current guidance takes vaccination into account so that couples seeking to conceive or in early pregnancy can be reassured.

In October/November 2020 Italy, especially Lombardy and specifically Milan, was struck by a second, more severe, pandemic outbreak. Nowadays, our internal protocols reckon on nasopharyngeal swabs checks only for women undergoing oocyte retrieval (OR), and the latter are performed 48 h before the procedure. Upon a positive test result, the OR is cancelled, the woman is taken care by her general practitioner and the cohabiting relatives quarantined until their oral COVID swabs results are available. On the other hand, as for the embryo transfer (ET) procedures, 48 h before a triage form is submitted in telephone communication by the medical staff of the Humanitas Fertility Center, in accordance with the European Society for Human Reproduction and Embriology (ESHRE) guidance on recommencing ART treatments (published April 23rd, 2020). If the patient gives an affermative answers to at least one of the questions, the nasopharyngeal swab is ordered and the ET postponed until a negative test. No antibody checking is contemplated.

Nowadays the positive rate in Lombardy is finally very low, 0.4% up to 24th June 2021, thanks to the excellent vaccination campaign started four months ago.

At the present time, a possible third wave is expected in our territory as well as worldwide due to the detection of new variants with potential for immune escape from vaccines. On the other hand, the first Italian COVID-19 vaccination report published by ISS on May 11, 2021^43^ and based on data collected on the first 13 million vaccinated in Italy, seems to be quite reassuring since the vaccine lowered the contagion by up to 80%, the hospitalization rate up to 90% and the death rate up to 95% already after the first dose. The vaccination plan represents in fact the only hope to cope with the dreadful SARS-COV2 virus and its variants for future years. Since herd immunity is yet to come, the goal of future global efforts to defeat the pandemic crisis is to reach a global vaccination coverage. The project is ambitious and difficult, but the world must not squander the opportunity.

As Yap et al. stated, a continuous update of robust evidence is needed to guide the gynecologists’ management of COVID-19 in pregnant women in order to keep pace with the emerging evidence during the pandemic^26^.

On this basis, Shah et al.^27^ showed that vertical transmission had occurred and interestingly explored all the possible ways of mother-to-fetus transmission of SARS-CoV-2. They stated that the infection during the first or second trimester may have the potential to cause miscarriage, preterm birth, birth defects or possibly other features of congenital infection, whilst in case of maternal infection in late gestation, one should consider the possibility that the newborn could have an active infection and consequently be at risk of adverse outcomes.

SARS and MERS epidemics showed no correlation with fetal malformations. However, the clinical course of COVID-19 disease and the response to treatments seem to differ from other previous types of coronaviruses^28^. In order to fully understand the pathogenesis and epidemiology of SARS-CoV-2 during pregnancy, further research is needed focusing on the time of maternal infection, gestational age, role of comorbidity factors, and adverse outcomes. During the first months of the pandemic, the American Society for Reproductive Medicine (ASRM) Coronavirus/COVID-19 Task Force reviewed ninety-seven articles on pregnancy and coronavirus in order to clarify the effect of the novel virus on human reproduction and pregnancy^11^. At the time, no reported studies examined pregnant COVID-19 patients at earlier stages of pregnancy. Few data regarding the effect of SARS–CoV-2 on human reproduction were available because the virus was novel and had only recently infected humans.

The SARS–CoV-2 virus enters human cells using ACE2 receptors. The reproductive system in men expresses ACE2 in Leydig cells in the testis and it may play a role in spermatogenesis. Gonadotropin-dependent expression of ACE2 has been reported also in human female gonads^29,30^, but it is still unknown if the SARS–CoV-2 virus uses ACE2 receptors in the human reproductive system and what, if any, impact this might have on oocyte quality, embryo development, or the consequent pregnancy.

. Concerning vertical transmission, from the early preliminary reports of few cases^31^, a number of systematic reviews and meta-analyses now available on this topic, conclude that it can occur in a minority of cases during the third trimester^8^. However, given the paucity of early trimester data, no assessment can yet be made regarding the rates of vertical transmission in early pregnancy and the potential risk for consequent fetal morbidity and mortality.

It is still unknown whether COVID-19 infection can impact fertility and human reproduction It is important to study long-term effects on male and female gametes, specifically whether there might be a shedding of virus in some individuals that might even affect the safety and storage of gametes^32^. While there are reports of SARS-CoV-2 viral particles being isolated in male reproductive fluids and tissue^33^, this has not been consistently demonstrated and more studies are needed before conclusive recommendations can be made regarding the handling of male reproductive fluid in men who have had COVID-19.

Evidence continues to emerge regarding effects of the novel coronavirus on pregnancy and reports suggest that complications, both in pregnancy^34^ and after delivery, may be increased^35^, even if outcomes for infants are largely reassuring when considering potential effects of SARS-CoV-2 infection acquired before or during birth^5,36^. The incidence of mother-to-newborn SARS-CoV-2 transmission appears low and may be related to biological, social or behavioral factors^37^. A novel condition named “pediatric inflammatory multisystem syndrome” has been temporally associated with SARS-CoV-2 infection^38^ and is still a matter of study to identify its optimal therapeutic approach. Further studies are necessary, and additional data regarding outcomes of early pregnancies in demonstrated infected pregnant women should be collected^39^.

Our study is an epidemiological cohort study on patients undergoing ART during the COVID-19 pandemic. Although limited by the general unavailability of SARS-CoV-2 testing results, it represents a functional overview of our internal ART cycles during this timeframe. We are aware that a significant, but unknown, number of our patients may have been asymptomatic carriers, but the emergency circumstances at the time did not allow testing. Our study represents an indirect evidence of two case–control studies (involving 46 patients and 287 controls) showing that COVID-19 during early pregnancy is not more severe than among non-pregnant women^40,41^. The strength of the present study relies on the large sample analyzed in a geographical area considered as the epicenter of the pandemic in Europe and where the incidence of SARS-COV-2 positive cases was very high in the general asymptomatic population.

The main limitation of this study is the retrospective nature of the analysis and the high plausibility (though not corroborated) of COVID-19 exposure since the ART treatments occurred during the early peak of the Italian pandemic.

## Conclusion

There is still a paucity of information about the impact, if any, of COVID 19 infection on early pregnancy (first trimester) outcome. Since it is very plausible that the novel SARS-COV2 virus could have spread, unrecognized, in the Lombardy area already few months before ART treatments were suspended, this study investigated whether the several infertile asymptomatic patients who were treated and achieved pregnancy during that at-risk time period had a different pregnancy outcome if compared to a similar infertile population dataset taken during the same season but one year before (non-COVID-19 risk).

Our dataset mitigated the concerns for negative reproductive consequences from COVID-19 pandemic and demonstrated no increased risk for miscarriage during the first trimester of ART pregnancies achieved during the emergence of the COVID 19 health crisis.
